# Identifying risk factors for Jihadist terrorist offenders committing homicide: An explorative analysis using the European Database of Terrorist offenders

**DOI:** 10.3389/fpsyg.2022.1000186

**Published:** 2022-11-23

**Authors:** Daphne L. Alberda, Nils Duits, Kees van den Bos, Anneke Autsema, Maaike Kempes

**Affiliations:** ^1^Department of Science and Education, Netherlands Institute of Forensic Psychiatry and Psychology (NIFP), Utrecht, Netherlands; ^2^Department of Psychology and School of Law, Utrecht University, Utrecht, Netherlands; ^3^Institute of Education and Child Studies, Faculty of Social and Behavioural Sciences, Leiden University, Utrecht, Netherlands

**Keywords:** convicted terrorists, homicide, European database, VERA-2R, risk assessment, comparative risk analysis, Jihadism

## Abstract

Although many studies have examined various aspects of terrorism, relatively little is known about risk indicators associated with specific types of terrorist offences. To partly fill this void, this study explores differences on risk indicators of the Violent Extremism Risk Assessment tool (VERA-2R) between 21 Jihadist offenders who were convicted for homicide and a comparison group of 30 Jihadist offenders convicted for other Jihadist terrorist offences. In doing so, we use judicial data from the European Database of Terrorist offenders (EDT). The results reveal that a number of risk and protective indicators differ between both groups. Both terrorist offender groups often expressed grievances about perceived injustice, but the homicide group more frequently expressed anger, moral outrage, or hatred in response to the perceived injustice than the comparison group. The homicide group also identified their attacks more often than the comparison group, and were more actively engaged in planning and preparation them. Additionally, the homicide group was less often motivated to commit their terrorist offences by group belonging compared with the non-homicide group. With respect to the protective indicators, persons in the comparison group more often reject violence as a means to achieve goals. Although further research is necessary, the results from this study indicate that a differentiated approach might be needed for risk assessment and risk management of the terrorist offender population.

## Introduction

Terrorism and violent extremism continue to pose significant security challenges worldwide, and also in Europe (Europol, 2022; Institute for Economics and Peace, 2022). European examples are the attacks in Berlin, London, Utrecht and Vienna. In 2016 a man drove into the crowd of people on a Christmas market in Berlin with a stolen truck. As a result of this act of terrorism, 12 people were killed and more than 50 people were injured. Another incident of homicide terrorism took place in 2017, when a man drove his van into the passers-by on the Westminster Bridge in London, killing five and wounding over 50 persons. In 2019, in the Dutch city of Utrecht a man opened fire on passengers in a tram, killing four people and severely injuring six others. In a terrorist attack in 2020 in Vienna, a man shot randomly at passers-by at six different locations in the center of the city, killing four people and injuring at least 15 others.

The ongoing threat of terrorism stresses the need to acquire more knowledge of causes for the engagement in these terrible acts. This empirically-based knowledge is key to better understand, predict, prevent, and disrupt terrorists’ actions (Knight et al., 2019). Given the great impact of terrorist attacks on societies, it is crucial to achieve an in-depth understanding of the mechanisms pushing individuals to perform such acts of homicide, in contrast to those who support terrorist movements but do not engage in homicide acts. This understanding can be improved when more is known about the personal drivers and motivations to commit specific terrorist acts. In this respect, grievances originating from feelings of injustice, are suggested to be a well-known driver for terrorism (Atran, 2003; Saucier et al., 2009; McCauley and Moskalenko, 2011; Monahan, 2012), although their association with specific terrorist acts remains unclear. Despite the rise in terrorist studies after the 9/11 attacks, empirical research into the similarities and differences of subgroups of terrorist offenders, among which the relevant differentiation between homicide versus non-homicide terrorist acts, is lacking (Knight et al., 2019). As a result, the terrorism research field and associated policy decision-makers lack solid insight into risk factors of terrorists who are actually willing and able to kill. Although the former group is just the tip of an iceberg of the fulfillment of multiple functions in terrorist movement, this group receives societies’ fullest attention, since these individuals commit the most dramatic crimes in terms of direct consequences (Horgan, 2008).

To address part of these problems, the present study aims to compare the prevalence of risk and risk mitigating indicators among terrorist offenders who actually committed homicide with those who committed non-violent terrorist offences. After all, one of the current challenges in terrorism research relates to the broad concept of terrorism, resulting in a great variety of definitions (Monahan, 2012; Pressman et al., 2018). Whereas terrorism generally is associated with the execution of lethal violence, a large part of the terrorist offenders is involved in other type of terrorist acts, such as financing, recruiting or supporting terrorist movements in other ways (Horgan and Taylor, 2011; Horgan et al., 2016; Perliger et al., 2016; Schuurman, 2020; Alberda et al., 2021; Duits et al., 2022). Because different terrorist offenses are likely to be accompanied by distinctive underlying risk factors (Monahan, 2012), it might be pivotal to assess an individual’s risk for terrorism in concordance with the type of terrorist offense the person committed.

Over the years, various violent extremism risk assessment tools have been developed, such as the Extremism Risk Guidelines 22+ (ERG 22+) and the Violent Extremism Risk Assessment (VERA-2R). Whereas the ERG 22+ is used in England and Wales, the VERA-2R is used worldwide by trained professionals in judicial practice for violent extremism risk assessment and risk intervention management (Pressman et al., 2018; Van der Heide et al., 2019). In the current study, we use the risk and protective indicators of the five main domains of the VERA-2R because the indicators consist of item and category descriptions and thus can be scored by the researchers.

The VERA-2R is based on the Structured Professional Judgment (SPJ) approach, which is used to integrate, combine and weigh relevant information on the risk indicators. The five domains and 34 included indicators originate from operational knowledge of law enforcement authorities involved in terrorism and national security analysts and empirical research. The first domain refers to beliefs, attitudes, and ideology. The indicators of this domain enable the identification of the belief system and corresponding emotions possibly causing a person’s support for using violence to further religious, political, social or other ideological goals. The second domain addresses the person’s social context and his or her intention to act. The included indicators are meant to distinguish supporters and sympathizers from those who want to use violence to achieve ideological goals. The third domain is relevant to an individual’s ability to plan and carry out violent extremist deeds. This can include a criminal history of violent acts, a violent extremist network, ideological training and/or organizational capacity. The fourth domain includes motivations identified as drivers of pushing an individual to violent extremism, whereby different motivations can be present at the same time. The fifth domain relates to six protective or risk-mitigating indicators. These indicators are important for identifying positive changes in persons, both at a specific and continuum point of time. Cultural and social contexts such as personal contacts, family, and close friendships can serve to encourage the individual to use violence to achieve ideological goals. Others are related to a reinterpretation of ideology and disengagement from terrorism (Horgan, 2008; Bjørgo and Horgan, 2009). Importantly, the relevance of each risk indicator may vary with the context of the individual (Pressman et al., 2018; Pressman and Duits, 2019). Based on the outcomes, different risk scenarios need to be formulated and substantiated with a risk management strategy for each of these scenarios (Douglas et al., 2014; Logan, 2014).

The evidence base of terrorism and violent extremism risk assessment can be improved with more empirical knowledge about risk differentiation. However, this knowledge is hampered by a lack of reliable data sets (Borum, 2015; Perliger et al., 2016; Knight et al., 2019; Schuurman, 2020), which has partly to do with methodological problems and practical limitations of obtaining detailed, comprehensive, and reliable data, and relevant information (Silke, 2001, 2009; Knight et al., 2019). To account for these concerns, we have constructed the European Database of Terrorist offenders (EDT; Alberda et al., 2021). This database is based on information from comprehensive judicial files, which contain relevant information about risk and protective factors for terrorism. The data set includes demographic data, childhood circumstances and mental health issues, together with the indicators of the Violent Extremism Risk Assessment tool (VERA-2R). Furthermore, different types of convicted terrorists are included, from far right to Jihadists as well as group actors involved in financing and lone-acting persons involved in lethal violence. Thus, in this paper we put forward the EDT as a relevant tool to acquire insight into risk factors of different terrorist offender groups.

The aim of this study is to build on the empirical foundation for risk differentiation in the terrorist offender population, as terrorism encompasses a broad spectrum of actors and actions. An optimal categorization distinguishes persons who are willing and capable to commit homicide, compared with those involved in supporting roles. To this end, we used a sample of the EDT data to compare VERA-2R indicators between Jihadist terrorist offenders convicted for homicide, or deceased after a terrorist act of homicide (hereafter: Jihadist homicide) and persons convicted for terrorist (Jihadist) offences with supporting roles. To our knowledge, this is the first empirical study comparing the main risk and protective indicators of an established terrorist risk assessment tool among two distinctive terrorist offender groups. Outcomes from this study could be used for an improved evidence-based approach of risk-differentiation regarding risk assessment and risk management of terrorist offenders.

## Materials and methods

### Materials

This explorative retrospective study uses a data set extracted from the EDT. The EDT data set contains more than 300 items relating to significant background variables for the risk of terrorism, among which all indicators of the VERA-2R (Alberda et al., 2021). This enables research into the relative importance of each indicator. The design of the EDT dataset is based on the scientific collaborative project ‘Tat-und Fallanalysen hochexpressiver zielgerichteter Gewalt’ (TARGET) that started in 2013 (Zick, 2017). TARGET was an interdisciplinary German research network about lone actors committing attacks in schools and public space. In relation to this, EDT items are derived from the NIFP codebook on lone actors (NIFP, 2013); the German Bielefeld/NIFP Codebook on Radicalization (Bielefeld/NIFP, 2015); the Lone Actor Codebook (Gill et al., 2014); the Profiles of Individual Radicalization in the United States (PIRUS) database (Jensen et al., 2020b); and the right-wing terrorism and violence dataset (RTV dataset) (Ravndal, 2016). These have subsequently been adjusted to violent extremists and terrorists in exchange with other researchers. Besides, a number of items are based on the extensive literature on violent extremism, terrorism and ordinary violence (Alberda et al., 2021). Finally, a number of items are explorative. The variables in the dataset mainly consist of option categories, but a couple of open text fields are included for additional in-depth information.

The information is derived from comprehensive judicial case files, containing qualitative information from court, prison and probation, police, intelligence agencies, public prosecutor, and forensic mental health assessments. Besides information about psychopathology, these assessments contain an extensive description of the person’s history and social context. Trained assessors from several European countries systematically have scored all available relevant information from the judicial sources into the EDT, with use of the EDT codebook.^1^^,^^2^ To diminish bias in coding between the different Member States due to various item interpretations, inter rater reliability analyses (IRR) of the individual coders are executed.^3^ For the strength of the agreement, Landis & Koch’s cut-off points were used (Landis and Koch, 1977). For the VERA-2R indicators reported in this article, the overall IRR is 0.71.

### Privacy and ethics

This study is approved by the Ethical commission of Leiden University in Netherlands.^4^ To comply with the European privacy regulations, the EDT data processing aspects were discussed by a committee of Dutch privacy and security advisors and presented to the Data Protection Officer of the Dutch Ministry of Security and Justice, which is the primary controller of the EDT. To protect personal data and to avoid traceability, these data fields are encrypted by a Trusted Third Party (TTP), meaning that all personal data in the EDT are pseudonymized.^5^ More information about privacy and security measures can be obtained from the first author.

### Sample

To create our research sample we extracted the complete sample of terrorist cases from the EDT, consisting of 213 individuals that are convicted for or deceased after a terrorist crime in Belgium, Austria, Germany, Sweden, or Netherlands between 2012 and 2021. This cross-border research sample allows us to examine the prevalence of several risk indicators, not related to a specific time or location. Although the EDT is developed to include all type of terrorist offenders, it mostly consists of Jihadist terrorist offenders. Because it is hypothesized that different terrorist subgroups may have different risk indicators (see for example: LaFree et al., 2018), analysing a heterogeneous terrorist population could lead to potential bias arising from group differences. To diminish the heterogeneity of the research sample, we selected male offenders, aged 18 or older at the time of their conviction for a Jihadist terrorist crime or their Jihadist terrorist suicide attack. This has led to the exclusion of 61 persons (19 female offenders, 24 offenders aged below 18 years, and 18 non-Jihadist offenders).

The aim was to create the largest possible distinction between the research group and the comparison group, so that the results would be most telling and meaningful in terms of differences between actual contrasting terrorist crimes. In order to reach this, it was pivotal that only “extremes” in acts would be included, avoiding the use of terrorist crimes which could belong in both groups, depending on motivation. Thus, in determining in which category a case file needed to be sorted, the nature of the committed crime was considered, based on the criminal code of the conviction. If an act involved hands-on violence, irrevocably resulting in the death of other persons, it was placed in the homicide group. Only murder and homicide belonged in this category without doubt. For our homicide group (HG), this resulted in the inclusion of 21 individuals who committed a homicide-related terrorist crime, namely (attempted) murder (*N* = 19) or manslaughter (*N* = 3). Thirteen persons in this group were convicted for a terrorist offence accompanied by deadly violence, as became clear of the corresponding criminal code, eight persons died as a consequence of their deadly terrorist offence. Murder was clear in these cases because these persons died as a consequence of their committed terrorist attack.

Of crimes such as threatening, participating in foreign military service, preparation of murder and attempted homicide, it could easily be questioned whether the act involved killing: that depends on a perpetrator’s intent and the course of his acts. Since this is unknown as they were only convicted for the latter, these crimes are no solid base to include in one the investigated groups. A similar reasoning exists while excluding crimes such as fire-setting and taking hostages: there is no evidence of lethal consequences. Therefore, to avoid the inclusion of individuals in this comparison group who were willing to kill, but were arrested before they could strike, we excluded persons who were convicted for preparatory terrorist acts, among which: training, threatening, participating in a terrorist organization and/or preparation. This resulted in the exclusion of 101 persons in which the intention of homicide remains uncertain.

Thus, for the comparison group crimes were selected that differentiated the most from homicide, being suitable since its perpetrators (seem to) stay far away from participation in lethal acts. This concerned involvement in violent extremist crime by means of incitement (*N* = 4), financing (*N* = 8), recruiting (*N* = 8), supporting (*N* = 9), unlawful possession of material (*N* = 2) and/or member of a terrorist organization (*N* = 15). Finally, to diminish the chance of including persons willing to kill in the comparison group, we excluded persons in this group if ‘subject was willing to kill’, ‘subject had weapons’, or ‘subject used weapons’ was coded with ‘yes’ in the EDT. This resulted in the exclusion of three persons, resulting in a final comparison group of 30 persons.^6^

Given that persons from the homicide group as well as from the comparison group are convicted for the involvement of a Jihadist terrorist offence, in this paper we refer to Jihadist offenders for both groups. However, actually they may not meet the original definition of a Jihadist, since they do not necessarily need to have a Jihadist ideology themselves or they did not express it.

### Variables and coding

The following demographic variables, which are all based on the last known situation prior to the terrorist crime, were included to describe the research groups: the mean age at the time of conviction or decease, including the minimum age, maximum age and standard deviation; the person’s relational status; the living situation; the highest started education, which is based on the International Standard Classification of Education (ISCED); together with a variable indicating if the person finished school; occupation; and migration background, which is defined in the EDT as: ‘Subject lives temporary or permanently in a country where he or she was not born’. ‘Former crimes’ is defined as a former police record or judicial record. Finally, the involvement of other persons in crime is described in the EDT as: ‘From judicial file can be derived that other persons are involved in the index crime and/or named in the file, regardless of whether persons involved in the index crime are all part of same terrorist group and if the other persons are convicted or not.’

The VERA-2R indicators each have item descriptions, which are based on the VERA-2R manual (see Table 1). Whereas the VERA-2R risk indicators in the instrument have the category options: low, moderate, high, the risk indicators are operationalized into the EDT with the following categories: 0 = No, documented, 1 = No, unlikely, 2 = Yes, likely, 3 = Yes, documented, −99 = Information fails, and-96 = Not applicable. This scale is used in the EDT for quality reasons, as it allows for distinguishing between levels of objectivity of information sources and to distinct between items that are explicitly or implicitly mentioned in the judicial file, with implicit information being coded as: 1 = No, unlikely or 2 = Yes, likely. For the current research the latter is trivial as the desired division is between an item being applicable, or not at all. Hence, the above-mentioned categories of the VERA-2R indicators were dichotomized in this study for analysing purposes. The values 0 and 1 were recoded into “no,” meaning that no information about the indicator was documented in the judicial file, and 2 and 3 were recoded into “yes,” indicating that the risk factor was (likely to be) present, based on the judicial information. The protective indicators in the EDT already are coded as “no” and “yes.” To allow for more understanding of the type of experienced grievances, in case of grievances of the subject, in a follow-up item the reason or origin of the perceived grievances can be described in a text field.

**Table 1:** The European Database of Terrorist offenders: Overview of the indicators of the VERA-2R domains.

**VERA-2R indicator**	**Description**
Commitment to ideology that justifies violence	Subject supports any ideology (political, religious, social or other cause) that justifies the use of violence to achieve ideological goals.
Perceived grievances and/or perceived injustice	Subject expresses any grievances that he/she and/or groups with which he/she identifies, are more deprived, oppressed or persecuted than they should be. The perceived grievances are related to political, religious, social or other issues. They do not have to be objectively true.
Dehumanization of designated targets associated with injustice	Dehumanization is the process by which one person or group views others as less than human, as animals, and regards them not worthy of humane treatment explicitly on the basis of the denied human qualities. During the Holocaust, the Nazis referred to Jews as rats. Subject describes enemies, victims or potential human targets as animals and/or denies their human qualities.
Rejection of democratic society and values	Any rejecting of values, norms or laws of democratic society in which subject lives. For example: promotes boycott democratic elections, espouses ideological standpoints rejecting democratically legitimized decisions or laws. Refuses to recognize internationally accepted legal governments.
Expressed emotions in response to perceived injustice	Any expression of anger, moral outrage or hatred in response to perceived injustice as individual or in terms of an identified group. Anger: Blaming/accusing people, threatening people, frightening people, or expressing feelings of revenge or vengeance. Moral outrage: Extreme passion and anger connected to a severe violation of moral principles. Hatred is a more intense emotion than anger or moral outrage.
Hostility to national identity	Any hostility of subject against national identity. Group loyalty of terrorists can lead to hostility towards national collective identity. Such hostility arises when personal and collective values are perceived as irreconcilable with national values.
Lack of empathy for those outside one’s own group	Any lack of empathy and/or understanding of subject for those outside own (cultural, religious or ideological) group.
Seeker, user or developer of violent extremist materials	Any involvement in development, communication, access to or dissemination of violent extremist or terrorist materials, other media and/or published or reproduced sources.
Target for attack identified	Any identification of a general (nonbelievers’, ‘capitalists’, or ‘the Eastern states’) or specific (person, group or location) target for violent extremist or terrorist attack mentioned in judicial information.
Personal contact with violent extremists	Any contact or association with individuals who engage in or support acts of violent extremism or terrorism. Not only related to index crime. Do not include family or longstanding friendships.
Expressed intention to commit acts of violent extremism	Any expressed intention to participate in acts of violent extremism or terrorism (to others, in social media, or in other media) This is inclusive intention of recruiting and other type of involvement in violent extremism.
Expressed willingness/ preparation to die for a cause/ belief	The willingness to die for the cause or belief to which he/she adheres is expressed for example by the intention to be a martyr or a suicide bomber.
Planning, preparation of acts of violent extremism	Any information on (interest in) planning and preparation of an act of violent extremism or terrorism in manifestos or other evidence. Also involvement in financing terrorist crime (for example financing can be only the distribution of money amongst warriors abroad), and/or preparations for traveling to battlefields are acts of violent extremism or terrorism.
Susceptibility to influence, control or indoctrination	Any signs of susceptibility to be influenced by a leader or person that advocates acts of violent extremism or terrorism. Information on circumstances where the subject is likely to be susceptible. Background information on prior susceptibility.
Early exposure to violence-promoting, militant ideology	Subject is exposed to political violence or pro-violence militant ideology from parents or other important persons during childhood or adolescence.
Network of family & friends involved in violent extremism	Subject has family and/or longstanding friends who have taken part in and/or support acts of violent extremism or terrorism. It is about long-standing friends. Do not include new violent extremist or terrorist ‘friends’ or ‘comrades’ or partner.
Violent criminal history	Convictions of any previous violent acts have to be included. Including public order crime with violence or property crime with violence
Strategic, paramilitary and/or explosives training	Subject has had any (para) military, or weapons or explosives training including online training, written materials or other tutorials before the terrorist crime or as part of the terrorist crime. This item is coded with ‘yes, likely’ if subject is convicted for traveling to battlefield.
Training in extremist ideology in own country or abroad	Subject participated in at least one training in extremist ideology and/or has been exposed to extremist leaders or has been influenced on the internet or from other media sources.
Organizational skills & access to funding / sources of help	This is about the ‘ability to carry out a complex plan, which requires funding (money), sources of help or assistance used to plan or prepare violent extremist or terrorist action of subject and/or others, and skills (delegating).
Motivated by perceived religious obligation/ glorification	Any interpretation of violent act being perceived as religiously driven obligation and/or act is glorified and highly praised by religious or higher authority (e.g., being considered as a religious martyr, martyr for the Islamic Umma).
Motivated by criminal opportunism	Any motivation by criminal opportunism (personal gain or financial benefit) to participate in violent extremist or terrorist acts.
Motivated by camaraderie, group belonging	Any motivation to participate in violent extremist or terrorist acts by friendship or camaraderie and/or the need to group belonging.
Motivated by moral obligation, moral superiority	Subject is motivated to take part in violent extremist or terrorist acts by his or her feeling or idea that his or her principles are important and/or superior to norms, values and/or principles held by others or the out-group. Moral principles and values are not religious principles (these are scored elsewhere): e.g., protection of children, collective cause (protecting nature).
Motivated by excitement and adventure	Any motivation to participate in violent extremist or terrorist acts by excitement, adventure or romance.
Forced participation in violent extremism	Any coercion to participate in violent extremist or terrorist acts by other persons.
Motivated by acquisition of status	Any objective documented motivation regarding the need for gaining status to participate in violent extremist or terrorist acts. For example, being a leader, being important and/or detained. This item is coded as ‘yes, likely’ if there are signs of willingness to gain status by showing off for example by making own propaganda or picture with flag of terrorist organization, mentioning the willingness to die as martyr.
Motivated by a search for meaning/ significance	An objective source shows that motivation to participate in violent extremist or terrorist acts is based on a search for meaning or significance in life. Meaning and significance in life is defined as the sense that one’s life is significant and purposeful. This is about a fundamental desire to matter, to be someone or to have respect.
Reinterpretation of the ideology	Subject demonstrates a change in ideological values about extremist and ideology, and considers moderation or new interpretation of his/her ideology. Any change in values or other interpretation of extremist ideology, for example stops contact with extremist friends, and no longer is involved in behavior related to violent extremist ideas.
Rejection of violence as a means to achieve goals	Any plausible reconsideration of use of violence to achieve ideological goals. This has to mentioned by objective source like probation worker.
Change in concept of the enemy	Subject changed his/hers concept of enemy and/or is open to reconsider concept of the enemy/ consider alternative viewpoints. Any reconsideration of concept of the enemy is mentioned by objective source.
Active Participant in programs against violent extremism	Subject is a active participant in one or more programs that promote non-violent acts in response to political, religious or ideological differences of opinion or perceived injustice.
Support from the community for non-violence	From judicial file can be derived that subject has support from community to relinquish the use of (extremist) violence, for example bringing him to programs against violent extremism or by talking about alternative views. The support does not only have to focus on the relinquishment of violence, but can also be general support, e.g., wanting to rebuild the relationship. Community or social network: a social group of people who share some characteristics, like living area or being member of a social club.
Support from family members for non-violence	From judicial file can be derived that subject has support from family members to relinquish the use of (extremist) violence, for example bringing him to programs against violent extremism or by talking about alternative views. The support does not only have to focus on the relinquishment of violence, but can also be general support, e.g., wanting to rebuild the relationship.

### Analysis

An anonymized data set was exported from the EDT and saved as SPSS file in a secured digital project map in the judicial network environment of Netherlands Institute of Psychiatry and Psychology (NIFP) from the Dutch Ministry of Security and Justice. The SPSS file was statistically analysed utilizing IBM SPSS version 27. To analyse the background characteristics in each group, descriptive statistics were executed. In addition, chi-square tests were performed to assess whether various variables (relational status, living situation, type of education, finishing school, occupation, migration background, criminal history and involvement of other persons in the terrorist crime) differed between the homicide group and the comparison group. Next, Fisher’s exact tests of independence were conducted to examine whether any differences exist in the presence of VERA-2R indicators between the homicide group and the comparison group. This nonparametric test suits studies with small sample sizes with two categorical variables, when the aim is to investigate whether the proportion of the first variable is different depending on the value of the second variable (McDonald, 2014). A two-tailed test was used because of the explorative nature of this study. Furthermore, an alpha of 0.05 was applied for all statistical analyses. Because of the exploratory nature of this study, we decided not to use strict probability values adjustments and hence perhaps miss possibly important findings (Rothman, 1990). Effect sizes were reported for the degree to which the two variables are associated with each other (Pallant, 2016). To measure the strength of the associations, Cramer’s *V* was used, which is an alternative to phi in 2 × 2 tables. For our interpretation of Cramer’s *V*, we follow Cohen (1988), who interprets a value <0.3 as small effect size (or weak association), values between 0.3 and 0.5 as medium (or moderate association) and > 0.5 as large (or strong association). Finally, we will provide some qualitative information regarding the origin of the perceived grievances, for both groups, which was based on the follow-up item of the presence of perceived grievances, in which the reason or origin of these grievances is described.

## Results

### Demographics and crime

Table 2 shows that individuals in both groups are on average 28 years old at the time of their conviction or decease (SD = 6). Furthermore, in both groups around the same percentage is married or is single. A significant difference between the groups is found in their living situation with most of the homicide group living with other terrorists in a house, whereas most of the comparison group living with their partner before the terrorist offence. With respect to the educational background, the largest part of the homicide group has a higher education. Although a larger part of the comparison group has a lower education, this difference turned out not to be significant, which also accounts for the percentage of offenders in both groups that finished their school. With respect to their occupation, one out of three persons in both groups is unemployed. Additionally, most of the persons in both groups have a migration background, which means that the person or one or both of his/her parents is/are born in a foreign country. Regarding the criminal history, two-third of the homicide group and half of the comparison group committed one or more other crimes prior to their terrorist offence. This includes a variety of crimes, from violence, property crimes with or without violence, public order crimes, to traffic related offences. Persons were rarely convicted for earlier terrorist crimes: only one person in the comparison group and two persons in the homicide group were convicted for a former terrorist offence. Finally, in both groups most offenders committed the terrorist crime with other persons. In the homicide group only four persons committed the crime without others. However, although acting alone, two persons likely have had contact with other violent extremists or terrorists, based on the corresponding VERA-2R risk indicator. In the comparison group seven out of eight lone acting persons in the comparison group had contact with other violent extremists or terrorists.

**Table 2:** Demographics, criminal history and type of terrorist crime in male Jihadist homicide offenders (HG) and male Jihadist non-homicide offenders (CG).

	**HG (*N* = 21)**	**CG (*N* = 30)**
	**Mean (Range; SD)**	**Mean (Range, SD)**
**Age (in years)**	28 (18–44; 6)	28 (19–43; 6)
**Relational status**	**%**	**%**
Single	38.1	40.0
Separated/ divorced	4.8	0
Partnered	0	10.0
Married	38.1	46.7
Missing	19.0	3.3
Living situation*		
Homeless	0	6.7
In parental house	9.5	26.7
Alone in house	14.3	6.7
With partner in house	9.5	40.0
With other family in house	4.8	3.3
With terrorists in house	47.6	3.3
With others in house	4.8	0
In Institution	4.8	0
Other	0	6.7
Missing	4.8	6.7
**Education**		
Lower (primary or secondary) education	38.1	46.7
Higher education	47.6	30.0
Missing	14.3	23.3
**Finished school**		
No	47.6	46.7
Yes	38.1	43.3
Missing	14.3	10.0
**Occupation**		
Unemployed	33.3	33.3
Student/Work	42.9	53.3
Missing	23.8	13.3
**Migration background**		
Yes, subject	47.6	50.0
Yes, one or both parents	14.3	30.0
No	38.1	13.3
Missing	0	6.7
**Former crimes**		
Yes	71.4	56.7
No	28.6	43.3
**Involvement other persons in crime**		
Yes	81.0	66.7
No	19.0	30.0
Missing	0	3.3

*p = 0.003 (Pearson Chi-Square, 2-sided).

### Beliefs, attitudes, and ideology

Comparative analyses regarding the first VERA-2R risk domain “Beliefs, attitudes and Ideology” (BA) are presented in Table 3. The findings demonstrate that in the homicide group, as expected, all offenders had an ideology that justifies violence, compared with 83% in the comparison group. However, these proportions were not significantly different. Although the proportion of offenders with perceived grievances did not differ significantly between both groups, a greater part of the homicide group expressed emotions of anger, moral outrage, or hatred in response to perceived injustice than the comparison group (resp. 52 and 17%, (*p* = 0.002), with Cramer’s *V* indicating a strong association (*V* = 0.650). This can be anger towards a particular person, a group, or an institution such as the government. An in-depth qualitative analysis revealed that offenders in the homicide group mainly mentioned *collective* grievances, injustice feelings and a strong sense of oppression against Muslims, with experiences of hatred as a result. For example, a person mentioned that all his life he has seen the blood of Muslims flow, and stated that he prays that God breaks the backs of those who oppose him. Also, they mentioned strong feelings of political and religious injustice and discrimination, along with the feeling that the West is waging a war against Islam. The comparison group on the other hand mostly mentioned *personal* grievances. These grievances were, for example, related to the absence of support for education, fewer changes in working place and negative experiences with the police. For example, the person expressed anger and feelings of revenge in response to perceived injustice about being fired unfairly.

**Table 3:** Beliefs, attitudes and ideology in male Jihadist homicide offenders (HG) and male Jihadist non-homicide offenders (CG).

**Beliefs, Attitudes and Ideology (BA)**		**HG (*N* = 21)**		**CG (*N* = 30)**			
		*N*	%	*N*	%	*p*	e.s.
Commitment to ideology that justifies violence	Yes	21	100	25	83.3	0.497	0.184
	No	0	0.0	2	6.7		
	Not reported	0	0.0	3	10.0		
Perceived grievances and/or perceived injustice	Yes	11	52.4	10	33.3	0.182	0.299
	No	1	4.8	5	16.7		
	Not reported	9	42.9	15	50.0		
Dehumanization of designated targets associated with injustice	Yes	5	23.8	3	10.0	0.664	0.125
	No	5	23.8	5	16.7		
	Not reported	11	52.4	22	73.3		
Rejection of democratic society and values	Yes	8	38.1	12	40.0	0.106	0.326
	No	1	4.8	10	33.3		
	Not reported	12	57.1	8	26.7		
Expressed emotions in response to perceived injustice**	Yes	11	52.4	5	16.7	0.002	0.650
	No	0	0	8	26.7		
	Not reported	10	47.6	17	56.7		
Hostility to national identity	Yes	5	23.8	6	20.0	0.193	0.313
	No	2	9.5	10	33.3		
	Not reported	14	66.7	14	46.7		
Lack of empathy for those outside one’s own group	Yes	10	47.6	8	26.7	0.166	0.338
	No	1	4.8	5	16.7		
	Not reported	10	47.6	17	56.7		

**p < =0.01 (*p* = two-tailed; e.s. = Cramer’s V).

Fisher’s exact tests were calculated on the valid N of categories Yes (yes, documented/ yes, likely) and No (no, documented/ no, unlikely). Mean IRR = 0.73 (range = 0.66–0.93).

### Social context and intention

As becomes clear from Table 4, four risk indicators from the domain ‘Social Context & Intention’ (SCI) are significantly more often present in the homicide group. The first risk indicator associated with homicide-related crimes, relates to the identification of a target for an attack, which can be a person, group, or location. This indicator was present in 86% of the cases in the homicide group, opposed to 37% of the cases in the comparison group (*p* < 0.001; *V* = 0.557). Furthermore, the homicide group more often expressed the intention to commit their terrorist act (67% vs. 40%; *p* = 0.005; *V* = 0.480), and/or expressed their willingness to die for their cause or belief (33% vs. 23%; *p* = 0.046; *V* = 0.408). Additionally, the data indicates that planning or preparing terrorist offences with a prevalence of 91% is highly associated with committing a homicide-related crime, compared with a prevalence of 50% in the comparison group (*p* = 0.040; *V* = 0.325). Apart from significant differences between groups, one other risk indicator stands out in terms of high rates in both crime groups: more than four out of five of the terrorist offenders in both groups had personal contact with violent extremists in an informal social context.

**Table 4:** Social context and intention in male Jihadist homicide offenders (HG) and male Jihadist non-homicide offenders (CG).

**Social Context & Intention (SCI)**		**HG (*N* = 21)**		**CG (*N* = 30)**			
		*N*	%	*N*	%	*p*	e.s.
Seeker, user or developer of violent extremist materials	Yes	12	57.1	26	86.7	1.00	0.010
	No	1	4.8	2	6.7		
	Not reported	8	38.1	2	6.7		
							
Target for attack identified***	Yes	18	85.7	11	36.7	<0.001	0.557
	No	0	0.0	11	36.7		
	Not reported	3	14.3	8	26.6		
							
Personal contact with violent extremists	Yes	18	85.7	25	83.3	0.572	0.124
	No	2	9.5	1	3.3		
	Not reported	1	4.8	4	13.3		
							
Expressed intention to commit acts of violent extremism**	Yes	14	66.7	12	40.0	0.005	0.480
	No	0	0.0	9	30.0		
	Not reported	7	33.3	9	30.0		
							
Expressed willingness/ preparation to die for a cause/ belief*	Yes	7	33.3	7	23.3	0.046	0.408
	No	2	9.5	14	46.7		
	Not reported	12	57.1	9	30.0		
							
Planning, preparation of acts of violent extremism*	Yes	19	90.5	15	50.0	0.040	0.325
	No	2	9.5	9	30.0		
	Not reported	0	0.0	6	20.0		
							
Susceptibility to influence, control or indoctrination	Yes	8	38.1	18	60.0	0.584	0.139
	No	2	9.5	2	6.7		
	Not reported	11	52.4	10	33.3		

*p < 0.05;

**p < 0.01;

***p < =0.001 (*p* = two-tailed; e.s. = Cramer’s V).

Fisher’s exact tests were calculated on the valid N of categories Yes (yes, documented/ yes, likely) and No (no, documented/ no, unlikely). Mean IRR = 0.82 (range = 0.66–0.93).

### History, action, and capacity

In the VERA-2R domain History, Action, and Capacity (HAC) only one of the risk indicators appeared to be associated with homicide. An early exposure to a violence-promoting or militant ideology is found in more than one out of three cases in the homicide group, as opposed to only one out of five cases in the comparison group (*p* = 0.027; *V* = 0.423) (see Table 5).

**Table 5:** History, action, and capacity in male Jihadist homicide offenders (HG) and male Jihadist non-homicide offenders (CG).

**History, Action and Capacity (HAC)**		**HG (*N* = 21)**		**CG (*N* = 30)**			
		N	%	N	%	*p*	e.s.
Early exposure to violence-promoting, militant ideology*	Yes	8	38.1	6	20.0	0.027	0.423
	No	3	14.3	15	50.0		
	Not reported	10	47.6	9	30.0		
Network of family & friends involved in violent extremism	Yes	9	42.9	11	36.7	0.477	0.166
	No	4	19.0	10	33.3		
	Not reported	8	38.1	9	30.0		
Violent criminal history	Yes	12	57.1	9	30.0	0.086	0.261
	No	9	42.9	20	66.7		
	Not reported	0	0.0	1	3.3		
Strategic, paramilitary and/or explosives training	Yes	12	57.1	10	33.3	0.508	0.143
	No	6	28.6	9	30.0		
	Not reported	3	14.3	11	36.7		
Training in extremist ideology in own country or abroad	Yes	12	57.1	10	33.3	0.124	0.323
	No	2	9.5	8	26.7		
	Not reported	7	33.3	12	40.0		
Organizational skills & access to funding/sources of help	Yes	16	76.2	19	63.3	1.00	0.038
	No	2	9.5	3	10.0		
	Not reported	3	14.3	8	26.7		

*p < 0.05 (*p* = two-tailed; e.s. = Cramer’s V).

Fisher’s exact tests were calculated on the valid N of categories Yes (yes, documented/ yes, likely) and No (no, documented/ no, unlikely). Mean IRR = 0.66 (range = 0.55–0.81). The inter-rater reliability of the last item (Organizational skills & access to funding / sources of help) had an inadequate value of 0.55. Therefore, the interpretation of this result have to be done with caution.

### Commitment and motivation

The fourth VERA-2R domain relates to the type of motivation(s) for the terrorist act. Table 6 shows that the motivation based on perceived religious obligation and/or glorification was relatively often reported in the homicide group compared with the non-homicide group (62% vs. 37%; *p* = 0.023; *V* = 0.427). On the contrary, motivation by camaraderie and group belonging was less often mentioned as (one of the) motivation(s) among the perpetrators of homicide-related crimes than among the perpetrators of non-homicide-related crimes (resp. 10 and 50%; *p* = 0.038; *V* = 0.476). Furthermore, in both offender groups it was rarely to never reported that the person was motived by criminal opportunism, by excitement, or that the person was forced into participation in terrorist acts.

**Table 6:** Commitment and motivation in male Jihadist homicide offenders (HG) and male Jihadist non-homicide offenders (CG).

**Commitment and Motivation (CM)**		**HG (*N* = 21)**		**CG (*N* = 30)**			
		*N*	%	*N*	%	*p*	e.s.
Motivated by perceived religious obligation/ glorification*	Yes	13	61.9	11	36.7	0.023	0.427
	No	1	4.8	10	33.3		
	Not reported	7	33.3	9	30.0		
Motivated by criminal opportunism	Yes	0	0.0	0	0.0	-	-
	No	14	66.7	17	56.7		
	Not reported	7	33.3	13	43.3		
Motivated by camaraderie, group belonging*	Yes	2	9.5	15	50.0	0.038	0.476
	No	4	19.0	3	10.0		
	Not reported	15	71.4	12	40.0		
Motivated by moral obligation, moral superiority	Yes	7	33.3	11	36.7	0.099	0.339
	No	1	4.8	11	36.7		
	Not reported	13	61.9	8	26.7		
Motivated by excitement and adventure	Yes	0	0.0	3	10.0	0.541	0.257
	No	4	19.0	10	33.3		
	Not reported	17	81.0	17	56.7		
Forced participation in violent extremism	Yes	1	4.8	1	3.3	1.000	0.027
	No	15	71.4	19	63.3		
	Not reported	5	23.8	10	33.3		
Motivated by acquisition of status	Yes	4	19.0	8	26.7	1.000	0.064
	No	2	9.5	3	10.0		
	Not reported	15	71.4	19	63.3		
Motivated by a search for meaning/ significance	Yes	1	4.8	8	26.7	1.000	0.080
	No	1	4.8	5	16.7		
	Not reported	19	90.5	17	56.7		

*p < =0.05 (*p* = two-tailed; e.s. = Cramer’s V).

Fisher’s exact tests were calculated on the valid N of categories Yes (yes, documented/ yes, likely) and No (no, documented/ no, unlikely). Mean IRR = 0.64 (range = 0.46–0.86). The inter-rater reliability of the item ‘Motivated by criminal opportunism’ had an inadequate value of 0.46. Therefore, the interpretation of this result has to be done with caution.

### Protective and risk mitigating indicators

The last VERA-2R domain consists of protective or risk mitigating indicators. Table 7 shows that three protective indicators are significantly less often present in the homicide group than in the comparison group. The first indicator, reinterpretation of the ideology, was significantly less often present in the homicide group (resp. 14 and 40%; *p* = 0.024; *V* = 0.376). The second indicator, rejection of violence as a means to achieve goals, significantly differs in proportion between groups as the indicator is present in 10% of the homicide group and 47% of the comparison group (*p* < 0.001). The strength of this association, as represented by the Cramer’s *V* effect size, was strong (*V* = 0.750). With respect to the indicator participating in programs against violent extremism, we found that relatively more active participants were present in the comparison group (27%) than in the homicide group (0%) (*p* < 0.001). This association appeared to be also strong (*V* = 0.609). Although the comparison group indeed could have been more willing to actively participate in judicial programs, it could be possible that these programs were more often offered to the comparison group. Therefore, this result may not indicate a real protective indicator. The last finding of interest refers to the support from family or other important persons to relinquish the use of violence. Although this indicator did not differ significantly between groups, almost half of the persons committing non-homicide had support from family or other important persons to relinquish the use of violence (47%), compared with 19% of the persons committing homicide.

**Table 7:** Protective and risk mitigating indicators in male Jihadist homicide offenders (HG) and male Jihadist non-homicide offenders (CG).

**Protective and Risk mitigating indicators (P)**		**HG (*N* = 21)**		**CG (*N* = 30)**			
		N	%	N	%	*p*	e.s.
Reinterpretation of the ideology*	Yes	3	14.3	12	40.0	0.024	0.376
	No	14	66.7	10	33.3		
	Not reported	4	19.0	8	26.7		
Rejection of violence as a means to achieve goals***	Yes	2	9.5	14	46.7	<0.001	0.750
	No	14	66.7	2	6.7		
	Not reported	5	23.8	14	46.7		
Change in concept of the enemy	Yes	1	4.8	5	16.7	0.165	0.348
	No	13	61.9	9	30.0		
	Not reported	7	33.3	16	53.3		
Active Participant in programs against violent extremism***	Yes	0	0.0	8	26.7	<0.001	0.609
	No	16	76.2	7	23.3		
	Not reported	5	23.8	15	50.0		
Support from the community for non-violence	Yes	2	9.5	3	10.0	1.000	0.091
	No	1	4.8	1	3.3		
	Not reported/not applicable	18	85.7	26	86.7		
Support from family members for non-violence	Yes	4	19.0	14	46.7	1.000	0.091
	No	1	4.8	2	6.7		
	Not reported/not applicable	16	76.2	14	46.7		

*p < =0.05;

***p < =0.001 (*p* = two-tailed; e.s. = Cramer’s V).

Fisher’s exact tests were calculated on the valid N of categories Yes (yes, documented/ yes, likely) and No (no, documented/ no, unlikely). Mean IRR = 0.67 (range = 0.42–0.87). The inter-rater reliability of the last three items had an inadequate value of 0.42–0.53. Therefore, the interpretation of these results have to be done with caution.

In sum, considering the VERA-2R risk indicators, we found seven indicators to be positively associated with Jihadist homicide (see Table 8). The findings of the first risk domain, showed that the homicide group more often expressed anger and/or hatred in response to perceived injustice. Furthermore, the Social Context and Intention domain showed that intentional risk indicators were more often present in the homicide group, among which the identification of a target for attack, expressed intention for their offence and the willingness to die for it and preparatory actions. Moreover, the History, Action and Capacity domain showed that an early exposure to violence-promoting militant ideology was more often present in the homicide group than in the comparison group. Regarding the motivational indicators, the homicide group more often was motivated by religious obligation/glorification, whereas the comparison group relatively often was motivated by group belonging. With respect to the protective and risk mitigating indicators, only negative associations with the homicide group were found, related to reinterpretation of the ideology, rejection of violence to achieve goals, and being an active participant in judicial programs against violent extremism.

**Table 8:** VERA-2R Indicators positively or negatively associated with Jihadist homicide.

**Domain**	**VERA-2R Indicators**	**HG (*N* = 21)**	**CG (*N* = 30)**		
		%	%	*p*	e.s.
BA	Expressed emotions in response to perceived injustice**	52.4	16.7	0.002	0.650
SCI	Target for attack identified***	85.7	36.7	<0.001	0.557
SCI	Expressed intention to commit acts of violent extremism**	66.7	40.0	0.005	0.480
SCI	Expressed willingness/ preparation to die for a cause/ belief*	33.3	23.3	0.046	0.408
SCI	Planning, preparation of acts of violent extremism*	90.5	50.0	0.040	0.325
HAC	Early exposure to violence-promoting, militant ideology*	38.1	20.0	0.027	0.423
CM	Motivated by perceived religious obligation/ glorification*	61.9	36.7	0.023	0.427
CM	Motivated by camaraderie, group belonging*	9.5	50.0	0.038	0.476
P	Reinterpretation of the ideology*	14.3	40.0	0.024	0.376
P	Rejection of violence as a means to achieve goals***	9.5	46.7	<0.001	0.750
P	Active Participant in programs against violent extremism***	0.0	26.7	<0.001	0.609

*p < =0.05;

**p < =0.01;

***p < =0.001 (*p* = two-tailed; e.s. = Cramer’s V).

## Discussion

This article aimed to increase the understanding of the association between risk indicators for terrorism and distinctive terrorist offenses, and specifically homicide and non-homicide related terrorism. To this end, we examined the presence of risk and risk mitigating indicators from the Violent Extremism Risk Assessment tool (VERA-2R) in an EU sample of 21 Jihadist offenders who were convicted for homicide and 30 Jihadist offenders who were convicted for other terrorist offences as a comparison group. Although grievances seem to be a risk indicator for terrorism in general, the results of this study revealed that especially anger about these grievances can be associated with homicide Jihadism. Besides, homicide can be associated with the identification of a target, expressed intention to commit the terrorist offence, planning or preparing acts and an expressed willingness to die for the cause or belief. Moreover, one risk indicator was negatively associated with Jihadist homicide: the motivation of a search for camaraderie or group belonging.

Interestingly, in contrast to risk factors being associated with the homicide group, protective factors for terrorism were more often found in the non-homicide group. Although the vast majority of both groups did support a violent ideology, the non-homicide group more often considers a moderation or a new interpretation of the ideology and rejects violence as a mean to achieve goals. They also relatively more often actively took part in programs against violent extremism.

Taken together, the results of our study support the notion that well-known risk and protective factors for terrorism and violent extremism differ between Jihadist offenders convicted for homicide and those convicted for supporting offences. This finding adds to earlier research suggesting terrorist within-group differences of potential risk indicators, among which negative life experiences, subsequent emotions and a low self-esteem (Knight et al., 2017), and economic and social loss of significance (Jasko et al., 2017). Furthermore, variables associated with violent subgroups relate to a lack of stable employment, the presence of radical peers, and having a criminal history (LaFree et al., 2018). Additionally, differences among terrorist subgroups are found based on the role and presence of psychopathology (Gruenewald et al., 2013; Corner and Gill, 2015; Gill and Corner, 2017; LaFree et al., 2018). Whereas these and other studies already showed that terrorist subgroups may have different risk-profiles, the current study found a variety of risk and protective factors for terrorism and violent extremism to be associated with persons convicted for Jihadist homicide.

Empirical knowledge about differentiation between terrorist offender groups based on their terrorist acts may have potential implications for terrorist risk assessment and risk management. For example, the findings from this study suggest that feelings of anger about perceived grievances may be a relatively important risk indicator given its possible association with Jihadist homicide. This finding is in line with the assumption that anger provides a greater willingness to adopt catastrophic violence (Pressman et al., 2018). Additionally, an emotion such as anger may increase risk-taking (Ayanian and Tausch, 2016), which can be a rather crucial element when committing homicide.

Regarding the type of grievances, a distinction can be made between personal and collective victimhood (Bar-Tal et al., 2009). In the current study, collective grievances were frequently mentioned in our homicide group, mostly related to the injustice of the offender’s Arab brothers and sisters experienced through Western industrialized nations. For example, some of them are convinced that Muslims are oppressed, unfairly treated and killed by people of other faiths all over the world. In this context Lankford (2018) describes that mass shooters and suicide terrorists did not only use experiences of individual suffering as a justification for killing people, but also the victimization of a larger group they identify with and feel connected to. Jensen et al. (2020a) even suggest that a perception of the community being harmed plays a major role in separating violent from nonviolent radicals. Related to this, Borum (2015) describes that inflicted harm on a group someone identifies with, can cause desire for revenge, since this is a way to express one’s grievances. Therefore, an important consideration could be that collective grievances may point to an increased risk for severe terrorist offences.

Another risk indicator that we found to be associated with Jihadist homicide, is the identification of a target, which already was hypothesized by Pressman et al. (2018), by suggesting that the more precise the target, the greater the chance of an attack. Furthermore, our results suggest that Jihadist homicide apparently often is accompanied by an expressed intention for it. Additionally, these acts need some preceding behaviour that indicates planning, preparation, and conducting research to engage in a violent attack, whereas supporting acts such as financing do not necessarily depend on prior preparatory activities. The expressed willingness or preparation to die for a cause or belief that we found to be associated with Jihadist homicide is in line with earlier research, which linked these expressions with mass murderers and violent acts involving the death of several persons (Saucier et al., 2009; see also Meloy et al., 2012, 2014). The planning of attacks with martyrdom as a goal therefore likely represent a higher risk and threat level (Pressman et al., 2018).

In the domain History, Action, and Capacity, an association was found with early exposure to a violence-promoting or militant ideology on the pathway to homicide related terrorist offenses. It is remarkable that the vast majority of the comparison group was not exposed to such ideologies, whereas it is a common risk factor in the homicide group. Those historical experiences of violence are known to be linked to increased aggression (Saucier et al., 2009), the internalization of hatred and a deeply rooted sense that violence is justified (Pressman et al., 2018). Possibly these mechanisms can also lead to extremely violent terrorist acts such as homicide.

With regard to a person’s motivation to commit a terrorist offense, religious motivations were mostly mentioned in the homicide group, whereas in the comparison group the aim to belong to a group served as main motivation for their non-violent supporting terrorist offenses. This could suggest that this psychological need of group belonging may not be an issue in Jihadist homicide. Finally, in contrast to risk indicators associated with the homicide group, protective factors for terrorism were rarely reported in cases of persons turning into Jihadist homicide.

### Limitations and future research directions

An important limitation of the current study is the small sample size. Besides, our comparison group was somewhat heterogeneous, as we had to combine different categories of terrorist offenses. In follow-up research it would be useful to further differentiate within the comparison group to the specific type of terrorist offenses. Additionally, due to the small sample size we were unable to analyse possible interactions between the indicators. Therefore, this explorative study is meant as a first step on which future studies can build to better understand the association between violent extremism risk indicators and violent or non-violent outcomes. Although the small sample size and the heterogeneity of the comparison group led to a lower change to detect relevant statistical significances, we did find significant differences regarding a subset of VERA-2R indicators. Further research is needed to understand the interactions between the different risk and protective indicators for terrorism, among which motivational and emotional factors, taking into account someone’s opportunity and capacities to commit terrorist offenses. As the number of included cases in the EDT is increasing, future research with the EDT could examine which combinations of risk indicators increase the change to engage in specific types of terrorism. Moreover, psychopathology is relevant to include in these analyses since it is suggested to interact with other risk indicators among which grievances (Duits et al., 2022). Although the VERA-2R has six additional indicators to assess the presence of mental disorders, future research on the EDT dataset could distinct between different types of disorders including traits and symptoms among different offender groups and their relation with co-occurring factors (Duits et al., 2022). An interesting distinction could be made between lone-actor terrorists and group terrorists, who are supposed to have different motivations (Knight et al., 2019) and psychopathology (Hewitt, 2003; Gruenewald et al., 2013; Corner and Gill, 2015; Gill and Corner, 2017). Whereas the current sample is small and mainly consists of group actors, future research with an expanded dataset is needed to examine differences in risk factors among lone-actors and group offenders, also with respect to the presence and types of psychopathology.

Another issue has to do with the distinction between terrorist offenders who did or did not use lethal violence. Of course, categorization of subgroups committing distinctive terrorist offences is a complex issue. First, murder is difficult to prove as terrorist offenders are often convicted for crimes in conflict zones. As a result, the number of persons who indeed executed lethal violence in reality will be higher than the amount of homicide offenders in this study, which we based on their conviction. Furthermore, the common glorification of violence of extremist groups makes it hard to differentiate between persons who only gave statements and those with real intentions to commit violence. We also note that a well-known complicating factor in terrorism research is that persons within terrorist organizations can be involved in different roles. For instance, some people do not change in terms of type of violence, whereas others migrate between various roles and activities over time and become more or less dangerous (Borum, 2011a,b; Horgan et al., 2016). Moreover, persons can have different roles in a terrorist organization, especially when the organizational structure is ambiguous (Schuurman, 2020). Although in our comparison group there was no proof of lethal terrorist violence, we therefore cannot rule out the possibility that persons in this group actually could have committed an attack in the future or would have been more violent if they had the capability and or opportunity. To partly address the above-mentioned issues, we followed recommendations of Schuurman (2020) to operationalize Taylor and Horgan’s (2006) distinction between event decisions and involvement. According to this notion for event decisions, people need to have showed clear intent, access to weapons and involvement in planning and preparatory activities among which target selection and logistics (Schuurman, 2020). On the basis of this we excluded those persons from the comparison group who possessed weapons, used weapons, or were willing to kill. Nevertheless, the possible mitigation of terrorist offender roles underlines the importance of future research to identify different pathways into terrorist engagement. However, the significant differences found in this study indicate that a distinction in risk and protective indicators can be made based on terrorist offenders who are convicted for homicide and those convicted for other terrorist offenses.

Another issue relates to missing information of VERA-2R indicators in judicial case files, including the pre-trial assessments. The reliability of this information depends on the process position of the person and his or her cooperation in interrogations and forensic mental health assessments, during which the suspect can withhold relevant information. Consequently, it appeared to be difficult to determine whether unreported VERA-2R indicators were actually absent risk indicators, or erroneously undocumented by the assessor or mental health expert. However, since this limitation accounts for the homicide group in the same manner as for the comparison group, we assume that the comparative results of this study will not be affected by this limitation.

Finally, we should note that the current study does not represent all types of terrorism across Europe, as our sample only considers convicted Jihadists and only includes Dutch, Belgian, German, and Swedish cases. Therefore, more research is needed to examine to what extent our findings extrapolate to other, non-religiously motivated terrorist offenders and other European populations of terrorist offenders. Ultimately, since the VERA-2R is used worldwide, it would be useful to replicate our EDT-based findings outside the EU to find out possible differences in risk and protective indicators among terrorist offenders from different parts of the world.

## Conclusion

Notwithstanding the above-mentioned limitations, we think it is also fair to note that this is one of the first comparative studies that examines how individuals convicted for Jihadist homicide compare with those convicted for supporting Jihadist offences regarding the well-known indicators from a violent extremism risk assessment tool. The use of primary source data, extracted from the European Database of Terrorist offenders makes it possible to analyse large amounts of adequate information and to draw some conclusions as to risk assessment pertaining to Jihadist homicide. Based on the findings of this study, differences in the presence of risk indicators within the terrorist offender population may be better understood. For practitioners it will have added value to have more insight into which (combination of) risk indicators have more relevance for which type of terrorist offenders. More specifically, insight into risk indicators for committing Jihadist homicide can have important consequences for terrorist risk assessment and risk management, given the severity of these offenses. Moreover, the significant findings among a number of protective indicators, such as the rejection of violence as a means to achieve goals, shows the relevance to also include protective and risk mitigating indicators, when assessing the risk of homicide and other forms of terrorism. We therefore hope that the explorative analysis we put forward here, using the VERA-2R indicators extracted from the EDT, may help researchers and practitioners to a more evidence-based assessment of the risk of homicide and other forms of terrorism.

## Data availability statement

The datasets presented in this article are not readily available because we have strong privacy rules related to data sharing, as the dataset originates from restricted judicial source information. Requests to access the datasets should be directed to NIFP, europa@dji.minjus.nl.

## Ethics statement

This study is approved by the Ethical Commission of Leiden University in Netherlands. To comply with the European privacy regulations, the EDT data processing aspects were discussed by a committee of Dutch privacy and security advisors and presented to the Data Protection Officer of the Dutch Ministry of Security and Justice, which is the primary controller of the EDT. To protect personal data and to avoid traceability, these data fields are encrypted by a Trusted Third Party (TTP), meaning that all personal data in the EDT are pseudonymized. More information about privacy and security measures can be obtained from DA. Written informed consent for participation was not required for this study in accordance with the national legislation and the institutional requirements.

## Author contributions

ND, DA, and AA contributed to the conception and design of the study. DA organised the database, performed the statistical analysis, and wrote the first draft of the manuscript. All authors contributed to manuscript revision, read, and approved the submitted version.

## Funding

The development of the EDT was funded for 2 years by the EU from 2017 (JUST-AG-2016-03, project number 763765) and a continuation of the EDT from 2020 (JUST-JCOO-AG-2020, project number 101007383). Additionally, this research was supported by the European Commission under Grant (Call JUST-JCOO-AG-2020, Project 101007383). The Dutch Counterterrorism Organization and the Dutch ministry of Justice and Safety funded the EDT partly in the period 2019–2022.

## Conflict of interest

The authors declare that the research was conducted in the absence of any commercial or financial relationships that could be construed as a potential conflict of interest.

## Publisher’s note

All claims expressed in this article are solely those of the authors and do not necessarily represent those of their affiliated organizations, or those of the publisher, the editors and the reviewers. Any product that may be evaluated in this article, or claim that may be made by its manufacturer, is not guaranteed or endorsed by the publisher.

## Footnotes

^1^The assessors have successfully completed a coding training program, in which expertise and experience is acquired in objective coding and knowledge of radicalization, violent extremism and terrorism. Interrater reliability was tested during this training to ensure consistency.

^2^The EDT codebook can be requested from the first author.

^3^Since the training cases had too little variation in the distributions of the ratings, kappa estimates appeared to be unrepresentatively low (Iftikhar, 2020). Therefore, an alternative kappa was used, based on the percentage of agreement between the coders, and corrected for agreement based merely on chance, given the number of answer options.

^4^The reference number of the ethical approval is: ECPW-2021/328.

^5^Data from the EDT contains highly sensitive and confidential information on individuals that are convicted for terrorist crimes. Therefore, measures were taken to comply with the prescribed procedures for such data and to safeguard principles of anonymity. The privacy and security measures of the EDT, such as encryption of personal data, are written down in the EDT Privacy Impact Assessment (Alberda et al., 2021).

^6^The final sample consists of cases from Belgium, Germany, Sweden, and Netherlands.
